# *Porphyromonas gingivalis*-induced glucose intolerance during periapical lesions requires its LPS throught a Th17 immune response

**DOI:** 10.1038/s41368-025-00403-6

**Published:** 2025-11-13

**Authors:** Sylvie Lê, Emma Sturaro, Charlotte Thomas, Thibault Canceill, Bertrand Ekambi, Nawel Fellouah, Claude Knauf, Anne Abot, Christophe Tenailleau, Benjamin Duployer, Pascale Loubieres, Alison Prosper, Swann Diemer, Rémy Burcelin, Franck Diemer, Matthieu Minty, Vincent Blasco-Baque

**Affiliations:** 1 Département d’Odontologie, Faculté de Santé, Université de Toulouse, Toulouse, France; 2 Service d’Odontologie Toulouse Rangueil, CHU Toulouse, Toulouse, France; 3 UMR1297 Inserm, Team InCOMM/Intestine ClinicOmics Metabolism & Microbiota, Institut des Maladies Métaboliques et Cardiovasculaires (I2MC), Université Paul Sabatier, Toulouse, France; 4 ABBIA GROUP, Abbia Data Sciences, Toulouse, France; 5 UMR1220 Inserm, Team Pathologies of the Gut-Brain Axis, Institut de Recherche en Santé Digestive (IRSD), Université de Toulouse, Toulouse, France; 6 Enterosys SAS, 57 rue de l’Innovation, Enterosys SAS, Labège, France; 7 CIRIMAT, Université de Toulouse 3 Paul Sabatier, INP, CNRS, Toulouse, France

**Keywords:** Bacterial pathogenesis, Diabetes complications

## Abstract

This study investigates the role of Interleukin 17 (IL-17) in exacerbating periapical lesions caused by *Porphyromonas gingivalis* (*Pg*) lipopolysaccharides (LPS) in the context of metabolic disease and its potential impact on glucose tolerance. Researchers developed a unique mouse model where mice were monocolonized with *Pg* to induce periapical lesions. After 1 month, they were fed a high-fat diet (HFD) for 2 months to simulate metabolic disease and oral microbiota dysbiosis. To explore the role of LPS from *Pg*, wild-type (WT) mice were challenged with purified LPS from *Porphyromonas gingivalis*, as well as with LPS-depleted and non-depleted *Pg* bacteria; IL-17 knockout (KO) mice were also included to assess the role of IL-17 signaling. The impact on bone lysis, periapical injury, glucose intolerance, and immune response was assessed. Results showed that in WT mice, the presence of LPS significantly worsened bone lysis, Th17 cell recruitment, and periapical injury. IL-17 KO mice exhibited reduced bone loss, glucose intolerance, and immune cell infiltration. Additionally, inflammatory markers in adipose tissue were lower in IL-17 KO mice, despite increased dysbiosis. The findings suggest that IL-17 plays a critical role in amplifying *Pg*-induced periapical lesions and systemic metabolic disturbances. Targeting IL-17 recruitment could offer a novel approach to improving glycemic control and reducing type 2 diabetes (T2D) risk in individuals with periapical disease.

## Introduction

Type 2 diabetes (T2D) is characterized by a chronic “low-grade” inflammatory reaction.^1^ Diabetic patients exhibit increased secretion of pro-inflammatory cytokines, disrupting insulin signaling pathways and contributing to insulin resistance.^2^ We were the first to demonstrate that such a mechanism was caused by lipopolysaccharides (LPS) translocating from intestinal Gram-negative bacteria to the bloodstream and toward host tissues, triggering thereby inflammation.^3^ In the light of the physiopathological mechanism observed in the intestine, we further observed that oral dysbiosis is present notably when induced by a fat-enriched diet inducing metabolic disease. Epidemiological data indicate that about 52% of the global adult population has experienced apical periodontitis (AP) in at least one tooth.^4^ Periapical diseases are bacterial infections affecting the periapical region of the tooth, accompanied by an immuno-inflammatory reaction involving all supporting tissues of the tooth, including bone and periodontal ligament. Diabetic patients, in particular, tend to experience increased tooth loss and more frequent endodontic treatments.^5^

However, the mechanisms through which LPS-*Pg* induces periapical disease, therefore representing a risk factor for T2D, remain unknown. LPS drives inflammation primarily through the Toll-like receptor 4 (TLR4) pathway, which regulates the innate immune response and recruits the adaptive immune system.^6,7^ Understanding how dysbiotic commensal bacteria contribute to chronic inflammation is crucial and notably the adaptative immune responses. Among the potential candidate mechanisms, key cytokines such as IL-1, IL-2, IL-22, and IL-17 could be involved. Within these adaptive responses, the literature suggests that IL-17 plays a pivotal role.^8,9^ IL-17 induces both local bone destruction and systemic metabolic disturbances through coordinated inflammatory responses. While its involvement in periapical lesions has been previously described, notably by Oseko et al.^10^, we hypothesize that *Pg* triggers IL-17–mediated immune and metabolic effects both locally and at distance throught its LPS. IL-17 is known for its role in autoimmune diseases and its interactions with the microbiota. For instance, reductions in Gram-negative bacteria correlate with a decrease in the Th17 response at both local and systemic levels.^11^ The differentiation and expansion of Th17 cells are partially controlled by certain LPS-producing anaerobic bacteria. We previously demonstrated in mice that dysbiosis-induced IL-17 production in mice is critical in promoting chronic inflammation and insulin resistance, notably through the reduction of Th17 cells in the ileum as demonstrated using RORgt knockout mice and transplanted germ free mice.^12^ Moreover, both human and rodent models have shown that bacterial DNA in adipose tissue acts as a risk factor for metabolic inflammation, further exacerbating T2D.^13^ This suggests that the role of the intestinal innate and adaptive immune systems, notably *via* IL-17, extends beyond the intestine towards peripheral tissues such as the liver, the pancreatic islets, and the adipose depots providing a causal role in intestinal dysbiosis.

We here extend the concept by proposing that the oral cavity represents additional significant source of dysbiotic bacteria, particularly in periapical diseases where IL-17 could play a role. Among the different bacterial candidates observed in oral dysbiosis during metabolic disease *Porphyromonas gingivalis* (*Pg*), a key oral pathogen, and its LPS are recognized as major contributors to periapical disease.^14,15^ The quantity of *Pg* in the oral cavity positively correlates with the severity of insulin resistance in diabetic mice. Our recent studies showed that metabolic disease is associated with the worsening impact of *Pg*.^13,14^ In humans, our previous research revealed a significant increase in *Pg* abundance in severe cases of periapical lesions with high Periapical Index (PAI = 5).^16^

To demonstrate the causal role of IL-17 in LPS-*Pg* induced periapical lesions in metabolic diseases, we have set up a unique and specific model of *Pg*-monocolonization inside the tooth in immune-competent mice while fed a fat-enriched diet. Features of oral disorders and glucose metabolism have also been investigated.

## Results

### Exploration of the potential role of *Porphyromonas gingivalis* in a cohort of patients characterized by different scores of granuloma severity

Periapical disease, mostly represented by granulomas, is a bacterial infection of the tooth’s endodontic area, characterized by a chronic immune-inflammatory response affecting the apical supporting tissues (bone and periodontal ligament). The severity of periapical lesion may be associated with a distinct microbial ecology influencing the inflammatory reaction. To diagnose the dysbiotic status of a human cohort we first quantified and characterized the 16S rDNA profiles by sequencing the microbiota from periapical lesions. Their severity was assessed using the Periapical Index (PAI), based on lesion size.

**Fig. 1:**
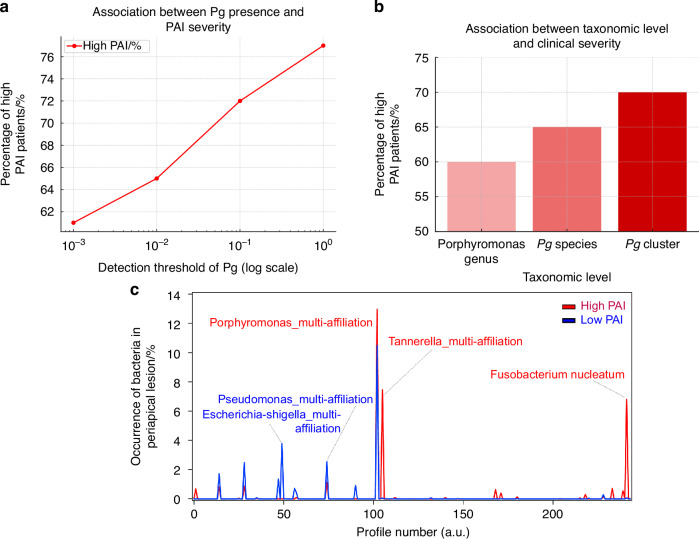
Exploration of the potential role of *Porphyromonas gingivalis* in a cohort of patients with varying granuloma severity scores. **a** Presence of the *Porphyromonas gingivalis* among patients with different PAI severity levels. The proportion of patients with high severity (PAI ≥ 5) and low severity (PAI ≤ 2) is shown in relation to the detection threshold of *Porphyromonas gingivalis* (logarithmic scale). **b** Association between *Pg* presence and PAI severity at different taxonomic levels. The relationship between the presence or absence of *Pg* and PAI severity is analyzed at the genus, species, and cluster levels. **c** Microbial composition in the presence of *Porphyromonas gingivalis*. Bacterial species detected in samples with a *Pg* detection threshold set at 1% are displayed. Species associated with higher severity are shown in red, while species associated with lower severity are shown in blue

As shown in Fig. 1a, the presence of *Porphyromonas gingivalis* (*Pg*) is associated with a more severe profile of periapical lesions, comparing microbial profiles between patients with severe lesions (PAI ≥ 5) and those with mild lesions (PAI ≤ 2).

We evaluated the presence of *Pg* based on detection thresholds ranging from 0.001% to 1% at the genus level. The results indicate that at a threshold of 0.001%, 42 patients had the *Porphyromonas* genus, among whom 61% had a high PAI. As the threshold increased, the percentage of patients with a high PAI, indicating a more severe periapical lesion, also increased. Thus, at a threshold of 1%, 77% of patients were associated with a severe clinical profile (Figure 1a).

Figure 1b shows, for a 1% threshold of *Pg*, the association between the presence of the bacterium and clinical severity at different taxonomic levels within the cohort. The more precise the analysis (from the genus level to the species level, then to the cluster), the higher the percentage of patients with severe clinical forms (PAI > 3), reaching 70% among patients with *Pg* presence above 1%.

Finally, Fig. 1c presents a species profile in the presence of *Porphyromonas gingivalis* with a detection threshold set at 1%. Three main peaks for High PAI patients (or for severe clinic forms) in red indicate the predominant associated gram-negative bacteria: *Porphyromonas* multi-affiliation, *Tannerella* multi-affiliation, and *Fusobacterium nucleatum*, in order of importance. What is also interesting to note in this Fig. 1c are the blue peaks (less severe bacteria) for Low PAI patients (or for non severe clinical forms) on top of the red peaks (severe bacteria), which could represent probiotics that fight against the severe bacteria for example Escherichia-Shigella multi affiliation.

In conclusion from Fig. 1, we could observe two distinct signature profiles depending on the granuloma pathology severity. In the High PAI patients we first notice the dominance of the Porphyromonas-Multi-affiliation species followed by the inflammatory Fusobacterium nucleatum and the periodontal associated Tannerella-Multi-affiliation species. In contrast, the low PAI profile was dominated by a distinct bacteria signature set essentially among Proteobacteria which can include some potential probiotic bacteria. So, we highlighted here a possible link between the granuloma severity, the *Pg* predominance and a possible synergy within a defined bacterial profile set for AP patients.

### A mutant LPS from *Pg* prevents *Pg*-induced periapical lesion, glucose tolerance, and local and systemic immune alterations via IL-17 production

The above results show a tight relationship between *Porphyromonas gingivalis*, present in periapical lesions, and the severity of these lesions. To study the causal role of LPS from *Pg* and IL-17 in the control of periapical lesions and glucose intolerance, we developed a relevant animal model. This model combines colonization with *Pg* (*Pg* WT), a strain mutated for LPS (No LPS Pg) or LPS purified from *Pg* (*Pg* LPS), with a high-fat diet (HFD).

We colonized C57Bl6 WT mice in the sterile endodontic cavity of the first molar. The mice were then fed with an HFD or NC for 2 months. This approach allowed us to combine the risk factor associated with *Pg* LPS with a key environmental factor of dysmetabolism in the study of the worsening of glucose intolerance induced by the HFD in the presence or absence of LPS from *Pg*. Additionally, we evaluated the relevance of *LPS from Pg* in immune cell recruitment, particularly to establish its role in the context of T2D.

**Fig. 2:**
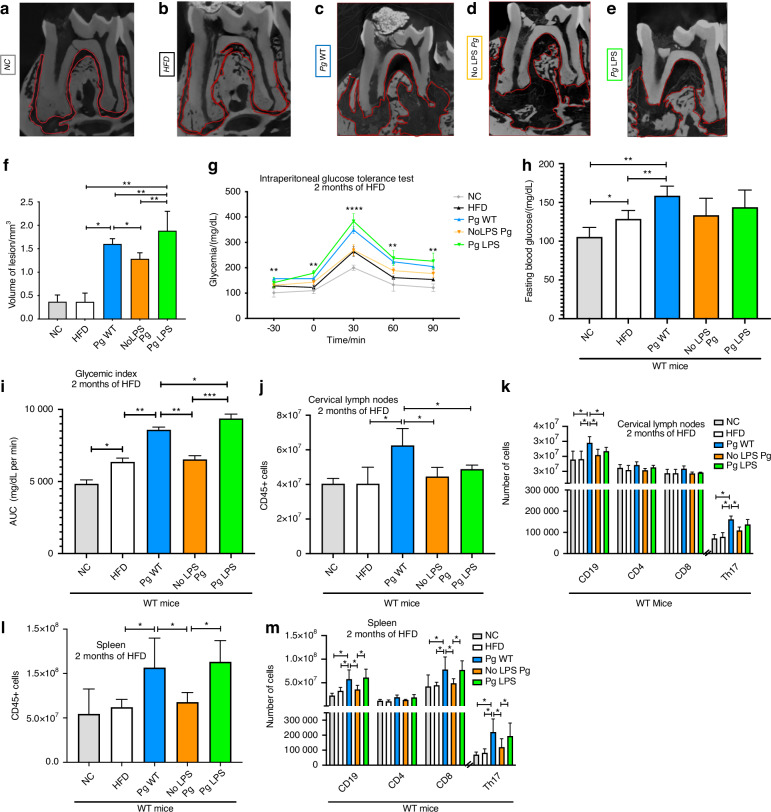
Impact of *Porphyromonas gingivalis* colonization and LPS on periapical lesions, glucose tolerance, and immune responses after 2 months of high-fat diet in wild-type mice. **a** Representative micro-CT sagittal section of the periapical region in control mice receiving normal chow (NC). No bone destruction is visible. **b** Representative images of HFD-fed mice without *Pg* colonization. The volume of periapical lesion remains minimal and comparable to NC mice. **c** Micro-CT analysis in mice colonized with wild-type *P. gingivalis* (*Pg* WT) under HFD reveals substantial periapical bone loss and large lesion volumes. **d** Representative images of mice colonized with the LPS-deficient *Pg* mutant strain (No LPS *Pg*) under HFD. Bone loss is markedly reduced compared to the *Pg* WT group. **e** Representative images of mice receiving purified *Pg* LPS under HFD. Lesion volume is comparable to that observed in *Pg* WT-colonized mice. **f** Quantification of periapical lesion volume (mm³) measured by 3D image analysis. No detectable lesions (N.D.) were found in NC and HFD-only groups. *Pg* WT and *Pg* LPS groups showed significantly higher lesion volumes compared to No LPS *Pg*. **g** Glucose curves from the intraperitoneal glucose tolerance test (IPGTT) performed after 2 months of HFD. Mice exposed to *Pg* WT or *Pg* LPS showed pronounced glucose intolerance compared to NC, HFD, and No LPS *Pg* groups. **h** Fasting blood glucose measured at *t* = –30 min. Mice colonized with *Pg* WT or treated with *Pg* LPS displayed significantly higher baseline glycemia. **i** Glycemic index represented by the area under the curve (AUC) of the IPGTT. Both *Pg* WT and *Pg* LPS groups exhibited increased AUC compared to other conditions. **j** Total number of CD45⁺ immune cells in cervical lymph nodes. The immune cell infiltration was elevated in the *Pg* WT and *Pg* LPS groups. **k** Analysis of immune cell subsets in cervical lymph nodes including CD19⁺ B cells, CD4⁺ T helper cells, CD8⁺ cytotoxic T cells, and Th17 cells after stimulation. The proportion of Th17 cells was significantly higher in mice colonized with *Pg* WT or treated with *Pg* LPS. **l** Total number of CD45⁺ immune cells in the spleen. The immune response was amplified in the *Pg* WT and *Pg* LPS groups compared to other groups. **m** Splenic immune cell subset analysis shows elevated numbers of CD19⁺ and CD8⁺ cells in mice receiving *Pg* WT or *Pg* LPS, along with increased Th17 responses. Color code: gray = NC, white = HFD, blue = Pg WT, orange = No LPS *Pg*, green = *Pg* LPS. Data are presented as mean ± standard deviation. Statistical significance is indicated as follows: *P < 0.05, **P < 0.01, ***P < 0.001, ****P < 0.000 1. Statistical tests used were Wilcoxon–Mann–Whitney for (**f**, **h**, **i**, **j**, **l**) and two-way ANOVA followed by Bonferroni’s post hoc test for (**g**, **k**, **m**)

Three-dimensional radiographic analysis (microCT) revealed that endodontic colonization with vehicle only, under both NC and HFD conditions (Fig. 2a, b, f; Supplementary Fig. 1), did not induce significant periapical lesions in terms of lesion volume, similar to non-operated sham controls. This suggests that diet alone is not sufficient to induce periapical pathology, and that colonization with *P. gingivalis* is necessary to trigger lesion development. In contrast, colonization with wild-type *P. gingivalis* (*Pg* WT) induced marked periapical bone lysis after 3 months, clearly illustrating a periapical lesion pattern (Fig. 2c, f). Colonization with the LPS-mutated strain reduces bone lysis and necrosis area compared to those induced by the wild type (WT) strain (Fig. 2d, f). For the group of mice that received purified *P. gingivalis* LPS (*Pg* LPS), we observed significant periapical bone lesions, with lesion volumes nearly comparable to those induced by *Pg* WT colonization (Fig. 2e, f). Thus LPS from *Pg* is therefore a major factor in the development of periapical lesions. It is noteworthy that we could not discriminate between the apical and the other regions, therefore, we considered the quantification of the overall region.

To complement the three-dimensional radiographic assessment of periapical lesions, we next performed histological analyses to characterize the local tissue responses induced by *P. gingivalis* strains at the cellular and extracellular matrix levels (Supplementary Fig. 3). While microCT provided quantitative evidence of bone resorption, histological examination enabled a more detailed evaluation of inflammatory infiltrates and fibrotic remodeling within the periapical region. Masson’s Trichrome staining of mandibular sections revealed that colonization with *P. gingivalis* WT induced marked periapical inflammation and collagen deposition compared to control groups (NC, HFD), highlighting the fibrotic nature of the lesions. Notably, infection with the purified *Pg* LPS mimicked the tissue alterations seen with the WT strain, whereas mice colonized with the LPS-deficient mutant strain (NoLPS *Pg*) exhibited attenuated histological responses (Supplementary Figs. 3 and 4).

To investigate the role of LPS during *Pg*-induced periapical injury on T2D parameters, 1 month after colonization, mice were fed a 72% HFD or NC. As shown in Fig. 2g, h, our results indicate that fasting blood glucose levels reveal a more pronounced diabetic phenotype in *Pg* WT-colonized mice after only 2 months of HFD compared to the control group (NC and HFD), despite individual variability in response to the diet.

Interestingly, mice colonized with the LPS-deficient mutant (in orange) or with purified LPS from *Pg* (in green) exhibit intermediate glucose levels between the control and *Pg* Wt-colonized groups, suggesting a potential role of LPS in promoting diabetes development (Fig. 2g, h).

After 2 months on the HFD diet, WT mice colonized with *Pg* WT show impaired glucose tolerance compared to mice not colonized on HFD or NC and mice colonized with No LPS *Pg* (Fig. 2g, i). Mice colonized with the LPS-mutated strain show similar glucose tolerance to mice not colonized under HFD (sham) (Fig. 2g, i). For the last group, *Pg* LPS, these mice show similar glucose tolerance than mice colonized with *Pg* WT (Fig. 2g, i).

In order to investigate the immuno-inflammatory disorders induced by endodontic *Pg* colonization, we assessed the quantitative and qualitative composition of immune cells locally in the cervical lymph nodes and systemically in the spleen after 2 months of HFD. No significant immune changes were observed between NC and HFD groups (control groups), indicating that HFD alone does not alter lymphoid immune cell composition in the absence of *P. gingivalis* colonization.

In the cervical lymph nodes of WT mice, colonization with *Pg* WT results in an increase in total immune cells (CD45+) (Fig. 2j) and Th17-profiled lymphocytes and a significant increase in CD19+ lymphocytes (B-cells) (Fig. 2k). Colonization with the LPS-mutated strain or with LPS purified from *Pg* prevents the recrutement of CD45+ and the increase in Th17 and CD19+ lymphocytes induced by endodontic *Pg* colonization (Fig. 2j, k).

In the spleen, *Pg* colonization in WT mice induces a significant increase in the number of CD45+ immune cells (Fig. 2l) and Th17-profiled lymphocytes (Fig. 2m). Colonization with the LPS-mutated strain prevents an increase in the number of immune cells (Fig. 2l), Th17 lymphocytes and CD19+ and CD8+ cells (Fig. 2m). Interestingly, purified *Pg* LPS alone was sufficient to increase total CD45⁺ splenocytes (Fig. 2l) and partially restore the expansion of CD19⁺ and Th17 cells (Fig. 2m), supporting the systemic immunostimulatory potential of LPS in the absence of live bacteria.

### The absence of IL-17 reduces periapical lesion formation and prevents local and systemic immune alterations leading to reduced glucose intolerance

**Fig. 3:**
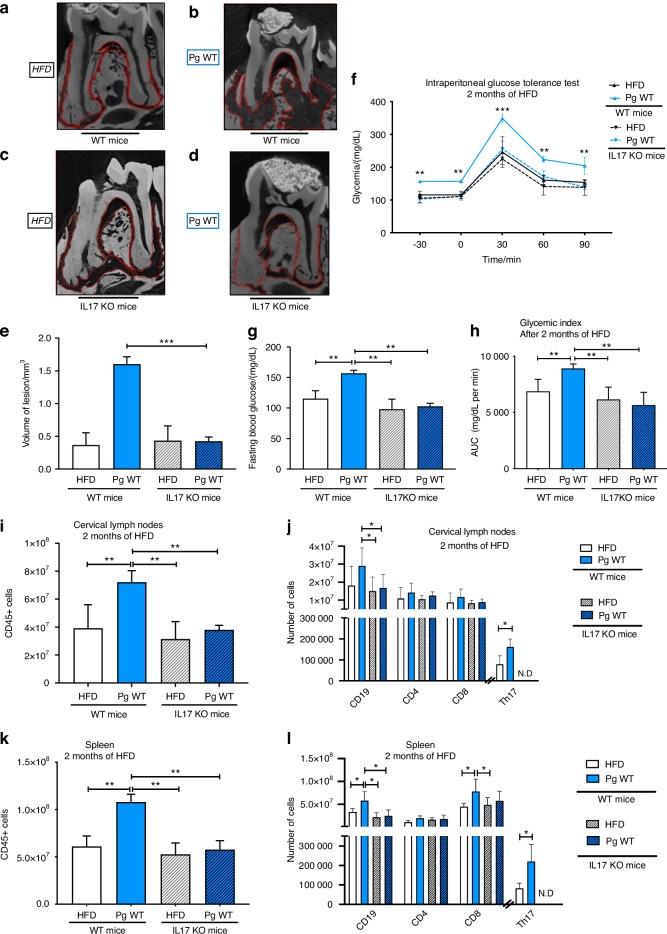
Impact of *Porphyromonas gingivalis* (*Pg* WT) colonization and IL-17 signaling on periapical lesions, glucose tolerance, and immune responses after 2 months of diet. **a**–**d** Representative micro-CT sagittal sections of periapical regions in WT and IL-17 KO mice under different conditions. **a** WT mice under HFD only. **b** WT mice colonized with *Pg* WT under HFD. **c** IL-17 KO mice under HFD only. **d** IL-17 KO mice colonized with *Pg* WT under HFD. **e** Quantification of periapical lesion volume (mm³). In HFD-only groups (WT and IL-17 KO), no significant lesion was detected (N.D.). *Pg* WT colonization significantly increased lesion volume in WT mice, and this effect was attenuated in IL-17 KO mice. **f** Intraperitoneal glucose tolerance test (IPGTT) performed after 2 months of HFD, showing glycemia over time. **g** Fasting blood glucose levels measured at *t* = −30 min of the IPGTT. **h** Glycemic index represented by the area under the curve (AUC) of the IPGTT. **i** Total CD45⁺ immune cell counts in cervical lymph nodes after 2 months of HFD. **j** Immune cell subsets (CD19, CD4, CD8, and Th17 after stimulation) in cervical lymph nodes. **k** Total CD45⁺ immune cell counts in the spleen. **l** Immune cell subsets (CD19, CD4, CD8, and Th17 after stimulation) in the spleen. Color code: White = HFD only (WT), Blue = *Pg* WT (WT), Striped white = HFD only (IL-17 KO), Striped blue = *Pg* WT (IL-17 KO), Gray = NC. Data are presented as mean ± SD. Statistical significance: *P < 0.05, **P < 0.01, ***P < 0.001*.* Statistical tests: Wilcoxon–Mann–Whitney for (**e**, **g**, **h**, **i**, **k**); two-way ANOVA followed by Bonferroni post-test for (**f**, **j**, **l**)

To further assess the causal role of IL-17 in *P. gingivalis*-induced periapical bone destruction, we conducted a loss-of-function experiment using IL-17 knockout (IL-17 KO) mice. Mice were colonized endodontically with *Pg* WT and maintained on a 72% HFD for 2 months. Three-dimensional radiographic analysis (microCT) were performed to evaluate periapical lesion volume (Fig. 3a–d; Supplementary Fig. 2).

As previously shown in WT mice, *Pg* WT colonization under HFD induced pronounced periapical bone lysis and necrosis (Fig. 3b). In contrast, IL-17 KO mice colonized with *Pg* WT exhibited a markedly reduced lesion volume (Fig. 3d), indicating that IL-17 is required for full lesion development. Quantitative analysis confirmed that lesion volume was significantly lower in IL-17 KO mice compared to WT mice (Fig. 3e), while no detectable lesions (N.D.) were observed in control HFD groups, regardless of genotype (Fig. 3a, c, e).

These results confirm that IL-17 is a key effector in *Pg*-induced periapical inflammation and bone resorption. The absence of IL-17 protects against periapical pathology, supporting its central role in mediating local immune-driven tissue damage following bacterial colonization.

To go further, we also reproduced the same experimental conditions used in WT mice—namely, colonization with the LPS-deficient *Pg* strain and administration of purified *Pg* LPS—in IL-17 KO mice (Supplementary Fig. 5a). Our results show that, in the absence of IL-17, neither the LPS-deficient strain nor the purified LPS alone were able to induce significant periapical lesions. This confirms that IL-17 is strictly required for the pathogenic effects of both live bacteria and their LPS component on periapical bone resorption.

Concerning histological analysis in IL-17 knockout (IL-17 KO) mice, periapical inflammation and fibrosis were significantly attenuated across all infection conditions. Regardless of the *Pg* strain (WT, NoLPS, or LPS), IL-17 deficiency conferred a protective effect, with consistently lower histological scores for both inflammation and fibrotic remodeling compared to WT counterparts, underscoring the central role of IL-17 in driving *Pg*-induced periapical pathology.

To investigate the role of IL-17 during *Pg*-induced periapical injury on T2D parameters we used IL-17 KO mice both treated with *Pg* WT and WT mice treated with *Pg* WT. As shown in Fig. 3f, g, our results indicate that fasting blood glucose levels are significantly higher in wild-type (WT) mice compared to IL-17KO mice after 2 months of HFD following colonization with *Pg WT*. This suggests that IL-17 deficiency may confer protection against diet-induced diabetes, as WT mice exhibit a more pronounced diabetic phenotype (Fig. 3f, g). Importantly, no significant differences were observed between WT and IL-17 KO mice in the HFD control groups (without *Pg* colonization), indicating that IL-17 does not significantly affect glucose homeostasis under diet alone, but specifically contributes to *Pg*-induced metabolic dysfunction. In IL-17 KO mice, none of the tested conditions—including colonization with *Pg* WT, the LPS-deficient strain, or administration of purified *Pg* LPS—resulted in significant metabolic alterations (Supplementary Fig. 5). Glucose tolerance remained stable across all IL-17 KO groups (Supplementary Fig. 5b), with no differences observed in glycemia curves following glucose challenge. Similarly, fasting blood glucose (Supplementary Fig. 5c) and glycemic index (Supplementary Fig. 5d) remained comparable between IL-17 KO mice and WT mice, regardless of *Pg* strain or LPS exposure. These results confirm that the absence of IL-17 protects against the systemic metabolic dysfunctions typically induced by *P. gingivalis* or its LPS in wild-type mice.

IL-17 KO mice colonized with *Pg* WT show an improvement in glucose tolerance compared to WT mice (Fig. 3f, h). These observations suggest that IL-17 is required for the impairment of glucose tolerance under HFD induced by *Pg* colonization as well as its LPS. Interestingly, IL-17 KO mice fed with HFD but not colonized with *Pg* displayed glycemic profiles similar to those colonized with *Pg* WT, indicating that HFD alone does not significantly impair glucose tolerance in the absence of IL-17 (Fig. 3f, h).

To investigate the specific role and contribution of IL-17 in the immune response, we assessed the quantitative and qualitative composition of immune cells locally in the cervical lymph nodes and systemically in the spleen at 2 months of HFD in IL-17 KO mice. Importantly, HFD alone did not lead to any major immune cell recruitment in IL-17 KO mice, either in lymph nodes or in spleen, indicating that the diet itself does not trigger significant immune activation in the absence of IL-17 (Fig. 3i–l).

In the cervical lymph nodes, the absence of IL-17 prevents the recruitment of CD45+ immune cells (representing total white blood cells) compared to WT mice in the same colonization (*Pg* WT) (Fig. 3i) associated with a significant decrease in the number of CD19 cells, representing B cells (Fig. 3j).

In the spleen, the absence of IL-17 prevents the recruitment of CD45+ immune cells compared to WT mice in the same colonization (*Pg* WT) (Fig. 3k) associated with a significant decrease in the number of CD19+ (Fig. 3l), and CD8+ lymphocytes.

**Fig. 4:**
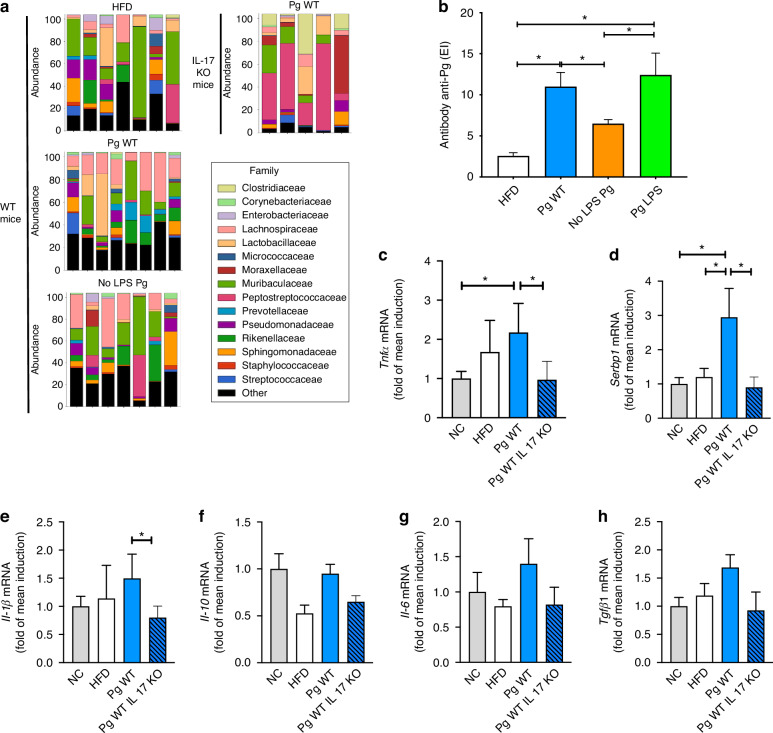
Bacterial translocation-induced metabolic inflammation in adipose tissue is driven by IL-17– dependent dysbiosis triggered by *Porphyromonas gingivalis* LPS. **a** Microbial composition of adipose tissue samples across different conditions (in WT mice three conditions Sham, *Pg* WT and No LPS *Pg* and in IL-17 KO mice only one condition *Pg* WT). Stacked bar plots representing the relative abundance of bacterial families in adipose tissue under different experimental conditions. Each bar represents an individual sample, and bacterial families are color-coded according to the legend. Only the top 15 most abundant families are displayed, the remaining families are aggregated in the “other” category. **b** Serum levels of anti-*P. gingivalis* antibodies measured by ELISA after 2 months of high-fat diet. Groups include HFD (vehicle), *Pg* WT, No LPS *Pg*, and *Pg* LPS. **c**–**h** Expression levels of inflammatory and fibrotic genes in adipose tissue measured by qPCR. Results are expressed as fold change relative to the NC group (normalized to 1). **c** shows *Tnfα* expression, **d** shows *Srebp1*, **e** shows *Il1β*, **f** shows *Il10*, **g** shows *Il6*, and **h** shows *Tgfβ1*. All gene expression data correspond to mice maintained under NC or HFD, colonized or not with *P. gingivalis*, in either WT or IL-17 KO backgrounds. Data are presented as mean ± SD. Statistical significance was determined using the Wilcoxon–Mann–Whitney test. Asterisks indicate *P* < 0.05. Color code: white = NC, gray = HFD, blue = *Pg* WT (WT mice), blue with stripes = *Pg* WT (IL-17 KO mice), orange = No LPS *Pg* (WT mice), green = *Pg* LPS

In addition, in IL-17 KO mice, neither colonization with the LPS-deficient strain (No LPS Pg) nor administration of purified *Pg* LPS led to significant changes in immune cell numbers in cervical lymph nodes (Supplementary Fig. 5e–f) or spleen (Supplementary Fig. 5g–h), further confirming that IL-17 is essential for both local and systemic immune activation driven by *Pg* or its LPS. Altogether, our results demonstrate that local and systemic Th17 cells are recruited by WT LPS, contributing to periapical diseases as well as the worsening of glycemic control. In contrast, mutant LPS prevents these mechanisms. The peripheral impact of oral and intestinal dysbiosis triggers systemic inflammation, notably in adipose tissue. To further investigate this inflammatory phenotype, we quantified various pro-inflammatory cytokines (Fig. 4).

### Metabolic inflammation induced by adipose tissue bacterial translocation depends upon LPS from *Pg* generated adipose tissue dysbiosis mediated by IL-17: Differential tissue microbiota and inflammatory responses in adipose tissue depend on *Porphyromonas gingivalis* colonization, LPS presence, and host genetic background (IL-17)

To investigate whether *Pg*-LPS-induced IL-17-mediated glucose intolerance in periapical disease is linked to adipose tissue metabolic inflammation and oral infection in adipose tissue associated with T2D, we quantified the impact of *Pg* on adipose tissue dysbiosis. We performed a series of analyses focusing on tissue microbiota compositions in mice colonized with *Pg*. The analysis of tissue microbiota composition (Fig. 4a) using 16S rRNA sequencing reveals distinct microbial profiles depending on the colonization by *Pg*. Tissue colonization with *Pg* WT induces a specific microbiota signature, which differs significantly from that observed with the LPS-deficient *Pg* strain and from HFD control mice. This suggests that *Pg* and its LPS contribute uniquely to shaping the tissue microbiota, with the LPS-deficient strain promoting an altered microbial profile.

The previous results are comforted by the observation that serum antibody levels were significantly higher of anti-*Pg* antibodies compared to mice colonized with the LPS-deficient *Pg* strain or HFD control mice in Fig. 4b. These findings also demonstrate that both *Pg* colonization and LPS presence shape tissue microbiota and inflammatory responses, while IL-17 plays a pivotal role in regulating *Pg*-induced tissue inflammation.

To further demonstrate the role of IL-17 in *P. gingivalis*-induced adipose tissue inflammation, we analyzed the expression of key inflammatory markers by qPCR in adipose tissue from WT and IL-17 knockout (KO) mice under different conditions (Fig. 4c–h). Colonization with *Pg* WT led to a significant upregulation of Tnfα, Serbp1, and Il1β mRNA expression in WT mice compared to NC and HFD controls (Fig. 4c–e). These increases were markedly reduced in IL-17 KO mice, indicating that IL-17 is required for the full transcriptional activation of these genes in response to *Pg* colonization. While Serbp1 is not a classical inflammatory marker, its regulation in this context may reflect broader post-transcriptional or metabolic changes induced by IL-17 signaling. In contrast, Il6 (Fig. 4g) and Tgfβ1 (Fig. 4h) expression showed a moderate but non-significant trend toward upregulation following *Pg* WT exposure, and Il10 (Fig. 4f) levels remained unchanged across groups. These findings suggest that IL-17 contributes to *Pg*-mediated adipose tissue alterations, notably through the induction of pro-inflammatory cytokines and potential post-transcriptional regulators such as Serbp1.

## Discussion

Within the frame of mechanisms explaining the epidemiological relationship between T2D and periapical diseases, we show that IL-17 is a causal factor responsible for the virulence of *Porphyromonas gingivalis* (*Pg*), establishing a mechanistic link that explains this epidemiological relationship. *Pg* has been identified in 30%–70% of root canal systems in teeth with endodontic diseases, emphasizing its key role as a major causative agent of periapical lesions.^17,18^ Our findings confirm its ability to induce significant periapical lesions, consistent with previous studies demonstrating *Pg’s* capacity to trigger local immune responses and tissue destruction.^19,20^ Importantly, our data extend current understanding by exploring the systemic implications of *Pg*-induced lesions, particularly their impact on metabolic health.

In the specific context of metabolic diseases, our results further demonstrate the causal role of *Pg* in metabolic risk and the worsening of metabolic conditions. Indeed, we show here that in HFD-fed mice, LPS from *Pg* exacerbates glucose intolerance. In this context of HFD, we observed that exposure to *Pg* and specifically to its LPS worsens glucose intolerance by targeting inflammatory mechanisms. These results are consistent with existing literature on other oral infections,^21^ which shows that periodontal infections increase circulating inflammatory markers and promote insulin resistance, especially when combined with HFD-induced metabolic stress.^21–23^ In addition, the bidirectional relationship between AP and glucose intolerance were studied as parallel conditions in epidemiologic studies.^24,25^ However the causal role of AP on glucose metabolism have not well described. Our findings support a causal link whereby *P. gingivalis*-induced periapical inflammation, especially *via* LPS and IL-17–mediated pathways, exacerbates systemic glucose intolerance. Our data show that LPS-*P. gingivalis* triggers a local Th17-mediated inflammatory response that promotes bone resorption.

Our findings also provide a deeper understanding of the mechanisms involved, highlighting the specific role of *Pg*’s LPS in metabolic dysfunction. The LPS of *Pg* plays a critical role in activating TLR4 pathways, triggering downstream inflammatory cascades involving cytokines such as IL-6, TNF-α, and IL-1β, which are known to exacerbate metabolic dysfunction.^26^ Additionally, LPS from *Pg* stimulates the recruitment of Th17 cells, which secrete IL-17, a cytokine essential for linking local inflammation to systemic metabolic disturbances. These mechanisms support the hypothesis that *Pg*-induced periapical lesions contribute to systemic metabolic diseases *via* these immuno-inflammatory pathways.^27–29^ We investigated whether IL-17 could acts as a molecular mediator linking *Pg*-induced periapical lesions to systemic metabolic dysfunction.

To investigate whether IL-17 acts as a molecular mediator linking *Pg*-induced periapical lesions to systemic metabolic dysfunction, we conducted experiments in mice lacking the IL-17 gene. Our results show that in these mice, the infusion of LPS from *Pg* failed to worsen glucose tolerance. To demonstrate the molecular causality of bacterial determinants, state-of-the-art experiments using germ-free mice were conducted, and to account for environmental factors such as the exposome—including nutritional risk factors like HFD-induced gut microbiota dysbiosis—we designed our experiments to include this dysbiotic dimension. This supports that AP contributes to local and systemic low-grade inflammation, disrupting the glucose parameters. Thus, in AP, oral infection driven by *Pg*, could represent a risk factor for metabolic disease. In this context, these phenotypes: bone loss, immune activation, adipose inflammation, and glucose intolerance, reflect a sequential cascade initiated by *Pg*-LPS. Local IL-17–mediated inflammation propagates systemically, inducing peripheral dysbiosis and cytokine release in adipose tissue, ultimately impairing glucose metabolism. This integrated mechanism supports the existence of an oral–systemic axis linking periapical infection to metabolic dysfunction.

To circumvent the mandatory dimension of gut microbiota dysbiosis and its relationship to periapical diseases, we established a unique model of colonization of germ free endodontic environement. Furthermore, to causally study a unique molecular hypothesis, we monocolonized this cavity with *Porphorymonas gingivalis*. In addition, to strengthen our demonstration, we colonized the mice with either a WT *Pg* or its LPS mutant. This approach highlighted not only the causal role of LPS from *Pg* but also the significance of its virulence factors, specifically the O-saccharide component of the LPS. This unique model shows that *Pg* LPS significantly recruits IL-17-producing cells both locally, within periapical tissues, and systemically, in distant organs. Using a mutant *Pg* strain with reduced LPS virulence, we observed attenuated periapical lesions and diminished systemic consequences. These findings are supported by prior studies in an intestinal context, where IL-17 exacerbated metabolic inflammation and impaired glucose metabolism.^30,31^ We contribute to our understanding of the role of IL-17 by demonstrating that, in the context of periapical lesions, IL-17 similarly mediates the adverse effects of *Pg* both locally and systemically. It is possible that IL-17 amplifies LPS signaling *via* TLR4 or recruits other pro-inflammatory immune cells, creating a feedback loop that worsens inflammation.^32^

However, the absence of LPS can trigger alternative inflammatory pathways, indicating that other virulence factors, such as fimbriae and gingipains, also contribute to the pathogenic effects of *Pg*. *Pg*, its LPS, and other virulence factors contribute to the invasion of periapical tissues, bacterial translocation into the bloodstream, and disruption of metabolic pathways.^19,33^ It will be of interest to further explore the synergistic interactions between *P. gingivalis* and other oral bacteria, such as *Fusobacterium nucleatum* and *Treponema denticola*. *F. nucleatum* invades tissues and activates TLR4, worsening systemic inflammation^34^ and insulin resistance, while *T. denticola* promotes tissue destruction and amplifies immune responses.^35^ Together, these pathogens contribute to oral dysbiosis and chronic inflammation, aggravating metabolic disorders.

Adipose tissue is implicated in diabetes through bacterial translocation. Indeed, we previously demonstrated that periapical disease induced by *Porphyromonas gingivalis* drives periodontal microbiota dysbiosis and insulin resistance *via* an impaired adaptive immune response.^21^ Additionally, adipose tissue inflammation has been consistently associated with excess fat mass and insulin resistance in both rodent models and in humans.^36^ Our study also reveals that periapical lesions can induce dysbiosis in adipose tissue, characterized by altered microbial composition and increased inflammatory profiles. While intestinal bacterial translocation is a well-documented driver of systemic inflammation,^3,37,38^ our findings suggest a similar role for periapical lesion-induced dysbiosis in promoting systemic metabolic dysfunction. IL-17 appears central to this process, as its absence significantly reduces the pro-inflammatory state and metabolic disturbances associated with *Pg*-induced adipose tissue dysbiosis. This aligns with previous studies implicating IL-17 in recruiting inflammatory monocytes and neutrophils, thereby exacerbating local and systemic inflammation.^39,40^ The oral epithelial barrier is essential for preventing pathogens and their products from entering systemic circulation. *Porphyromonas gingivalis* (*Pg*) disrupts this barrier through virulence factors like gingipains, which degrade junctional proteins such as E-cadherin, increasing permeability.^41–43^ This facilitates bacterial translocation and the release of microbial components like LPS into the bloodstream, triggering systemic inflammation.^44^ Similar to intestinal barrier dysfunction, this process links oral infections to systemic conditions such as glucose intelorance and cardiovascular diseases.

Overall, our results highlight the deleterious effects of *Pg* and its LPS on periapical lesion and metabolic diseases. We also reinforce the idea that these deleterious effects manifest through microbial dysbiosis of adipose tissue. These effects are significantly reduced in the absence of IL-17, underscoring its critical role as a molecular mediator in these processes. Furthermore, the interaction between IL-17, adipose tissue inflammation, and microbial dysbiosis offers promising therapeutic perspectives. Clinical studies show elevated systemic biomarkers like CRP, IL-6, and TNF-α in periodontitis and diabetes.^45^ Similar increases may occur in individuals with periapical lesions induced by *Pg* driven by inflammation from *Pg’s* virulence factors. Measuring these biomarkers could clarify the systemic impact of periapical infections and their link to metabolic diseases. The clinical implications of this study pave the way for innovative therapeutic approaches to mitigate the systemic effects of *Pg*-induced periapical lesions. Beyond targeting IL-17, strategies such as the use of gingipain inhibitors, key proteases of *Pg*, could help limit tissue destruction and bacterial translocation. Modulating oral dysbiosis with probiotics or prebiotics may also restore microbial balance and reduce systemic inflammation. Combined with conventional treatments, these interventions could alleviate metabolic disruptions and prevent associated systemic complications.

**Fig. 5:**
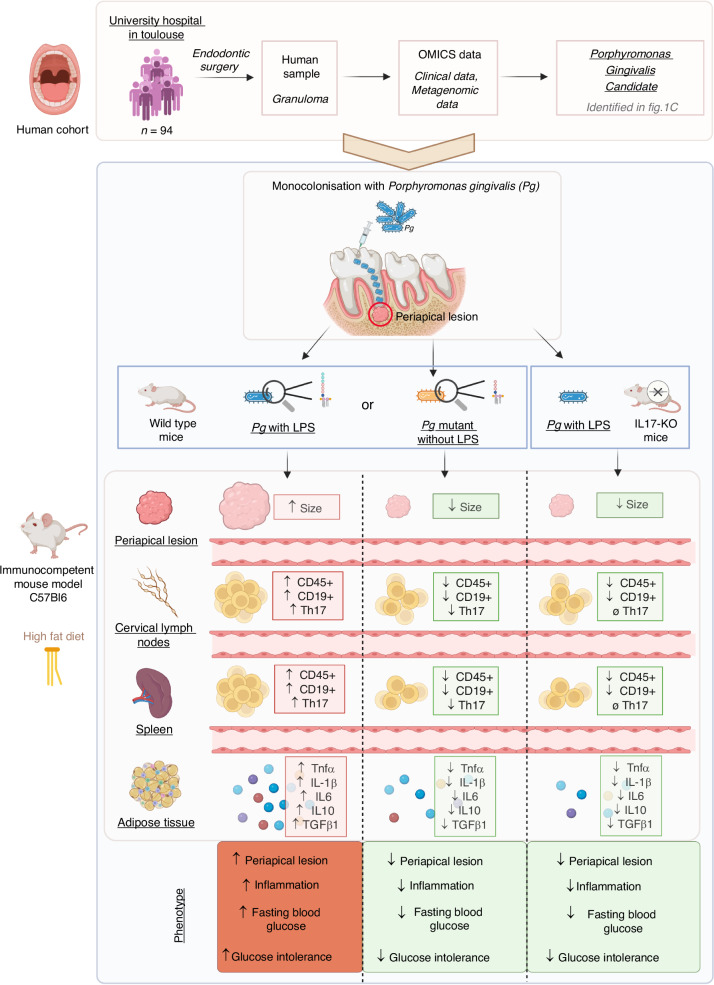
IL-17 mediates *Pg*-driven periapical inflammation and systemic metabolic dysfunction. Schematic overview of the experimental approach and key findings. Granuloma samples from a human cohort (*n* = 94) undergoing endodontic surgery at Toulouse University Hospital were analyzed using OMICS approaches, leading to the identification of *Porphyromonas gingivalis* (Pg) as a candidate pathogen associated with periapical lesion. Pg strains (wild-type with LPS or mutant lacking LPS) were used to mono-colonize immunocompetent C57Bl/6 mice fed a high-fat diet. *Pg* WT–colonized WT mice exhibited increased periapical lesion size, enhanced immune infiltration (CD45⁺, CD19⁺, Th17⁺) in cervical lymph nodes and spleen, and elevated pro-inflammatory cytokines (TNF-α, IL-1β, IL-6, IL-10, TGF-β1) in adipose tissue, leading to hyperglycemia and glucose intolerance. In contrast, both LPS-deficient *Pg* infection and IL-17 deficiency attenuated local inflammation, limited lesion progression, and improved metabolic outcomes

To provide a comprehensive overview, we summarize our proposed model and major findings in a synthetic schematic representation (Fig. 5), illustrating the sequential pathophysiological events from endodontic infection to systemic metabolic impact. Based on a human cohort of 94 patients undergoing endodontic surgery, metagenomic analyses identified *Pg* as a potential pathogenic candidate in periapical granulomas. Using a unique mouse model of endodontic *Pg* monocolonization combined with a HFD, the mice colonized with wild-type *Pg* (with its LPS) exhibited enlarged periapical lesions, increased infiltration of CD45⁺, CD19⁺, and Th17⁺ cells in lymphoid organs, and elevated expression of pro-inflammatory cytokines (TNF-α, IL-1β, IL-6, IL-10, TGF-β1) in adipose tissue, leading to impaired fasting glucose and glucose intolerance. In contrast, mice colonized with *Pg* lacking LPS, or IL-17 KO mice, were protected from both periapical bone loss and metabolic alterations—regardless of LPS exposure—thereby confirming the essential role of IL-17 in mediating the observed pathology. We show that the *Pg*-LPS–IL-17 axis orchestrates a cascade of local and systemic inflammatory events that culminate in HFD-induce glucose intolerance. Our findings establish IL-17 as a central mediator linking local Th17-driven periapical inflammation to systemic metabolic alterations, particularly through its effects on immune cell recruitment and inflammatory signaling.

To conclude, this study demonstrates that *Porphyromonas gingivalis* (*Pg*) and its lipopolysaccharide (LPS) are not only critical drivers of periapical bone destruction, but also key contributors to systemic metabolic dysfunction through TH17 immune response.

## Materials and methods

### Human collection: study population and sample collection procedures

The study reported here was approved by the Commission Ethique du Département de Médecine Générale de Midi Pyrénées in June 2016 and the Minister of Health (France), by the collection number N° DC-2022-5010. The STROBE statement guidelines for reporting were followed. Clinical samples were obtained from a cohort of 94 adult patients undergoing endodontic microsurgery for persistent AP at Toulouse University Hospital, as part of a translational research program coordinated by the INCOMM team (Inserm UMR1297). All participants provided written informed consent prior to inclusion. The inclusion criteria were: indication for surgical management of chronic AP, absence of systemic diseases other than T2D, no antibiotic use within the preceding 3 months, and availability of sufficient apical granuloma tissue for analysis. Clinical, anthropometric, and oral health parameters were collected using standardized questionnaires and clinical records. During surgery, periapical granulomas were collected aseptically, placed in sterile DNA-free cryotubes, snap-frozen in liquid nitrogen, and stored at −80 °C until analysis. DNA extraction from granuloma tissue was performed using a bead-beating protocol followed by purification with the QIAamp DNA Mini Kit (Qiagen), optimized for low-biomass clinical samples. DNA yield and quality were assessed using NanoDrop and Qubit. The V3–V4 hypervariable regions of the 16S rRNA gene were amplified using primers 341F and 805R. Sequencing was performed on the Illumina MiSeq platform (2 × 300 bp paired-end reads, v3 chemistry). Sequence processing was carried out using the DADA2 pipeline, with taxonomic annotation based on the SILVA v138 database. Relative OTU abundances were calculated per sample.

### Animals

Female C57Bl/6J, WT and IL-17 KO mice, 8 weeks old and grouped in cages of 6 were raised in a controlled non-pathogenic environment (at 22 °C, 12-h reverse day/night cycle with light off at 10:00 am) with free access to water and food (I2MC institut, Rangueil Toulouse, France).

The 8-week-old mice were operated under general anesthesia (ketamine solution, xylazine). Using dental surgery equipment, a hole is drilled in the first mandibular molars (right and left) to access the closed and sterile endodontic cavity. We deposit in this cavity 1 mL of solution of our choice contained in carboxy-methylcellulose. Thus, according to the deposited solution, the mice were randomized into five groups in each of the two categories of mice (WT and IL17 KO): Two groups did not undergo colonization (Normal Chow Diet (NC) and High Fat Diet (HFD)), these control mice received the vehicle only. The other three groups underwent colonization with either a WT *Pg* strain (WT) (ATCC 33 277), a purified LPS from *P. gingivalis (Pg LPS)*, or a mutated *Pg* strain whose LPS is devoid of its O-saccharide part (wzy: Em PGN_1242). The LPS-deleted strain was constructed as follows: a cassette conferring clindamycin resistance was inserted into a plasmid at the PG1242 region encoding the wzy O-polymerase, and this plasmid was then transfected by electroporation into the wild-type strain of *Pg*. Wzy is therefore no longer expressed and no longer allows polymerization of the O-polysaccharide part of the O-antigen forming the antigenic part of the LPS (Division of Microbiology and Oral Infection, Department of Molecular Microbiology and Immunology, Nagasaki University Graduate School of Biomedical Sciences, Nagasaki, 852-8588, Japan).^46^

The cavity was then hermetically sealed with a conventional endodontic cement. One month later, the uolonized mice (NC and HFD), the groups colonized with the wild-type *Pg* strain (*Pg* WT), the LPS-depleted strain (No LPS *Pg*) or a purified LPS from *P. gingivalis (Pg LPS)* were put on HFD or NC diets for 2 months. All animal experimental procedures were approved by the local ethical committee of the University Hospital of Rangueil (Toulouse) and INSERM (C3155507 and N°2020090315484518).

### Diet

The mice are fed with a Normal Chow Diet (NC), energy content: 12% fat, 28% protein and 60% carbohydrate; A04, Villemoisson-sur-Orge, France, or with a special team diet called the High Fat Diet 72% (HFD 72%) (for 2 months). The characteristics of this diet are: 72% lipids, <1% carbohydrates, and 28% protein in caloric equivalent. This HFD has the particularity of being non-obesogenic, and has the advantage of making the mice diabetic.^21^

### Validation of the model

#### Radiographic analysis

The left mandibles were removed on the day of sacrifice and stored at 4 °C until radiographic analysis. These analyses were performed by X-ray microtomography with a Phoenix/GE Nanotom 180 instrument from the French FERMAT federation (CIRIMAT, Toulouse, France). Each mandibule was put vertically in a tube positioned at a distance of 45 mm from the X-ray source. A C7942SK-05 Hamamatsu detector (2 300×2 300 pixels) was put at 300 mm from the source. Voltage of 80 kV and intensity of 180 μA were used. Voxel size was 7.5 μm. 1 440 sets of images were recorded over the complete sample rotation (i.e., 0.25° angle step scan) with 5 images of 750 ms each per step, while the first image was systematically removed in order to avoid reminiscence. DatosRec software was used for data reconstruction and VgStudioMax 2.1 was used for 2D and 3D data treatment.

#### Histological analysis

Right hemi-mandibles were excised post-mortem and immediately fixed in 4% paraformaldehyde or 10% neutral-buffered formalin (pH 7.4) for 48 h at 4 °C. After fixation, samples were decalcified in 10% disodium EDTA (pH 7.4) at 4 °C under gentle agitation for 3–6 weeks, with regular changes of the solution. Completion of decalcification was confirmed by radiography or mechanical testing. Tissues were then dehydrated through a graded ethanol series, cleared in xylene, and embedded in paraffin wax at 58 °C. Serial transverse sections (4–7 μm thick) were obtained using a rotary microtome along the longitudinal axis of the molars, encompassing the crown, roots, and periapical region. Sections were stained using Masson’s Trichrome protocol (Goldner or Heidenhain variant) to assess tissue architecture, highlighting nuclei (dark brown/black), cytoplasm and dentin (red), and collagen-rich structures (blue or green). Images were acquired using a slide scanner microscope. Histological analysis focused on representative regions of the periapical area. Inflammatory infiltrates and fibrotic remodeling were evaluated semi-quantitatively by two blinded independent observers using a 4-grade scale (from + to ++++) as illustrated in Supplementary Figs. 3 and 4.

### Analysis of metabolic parameters

#### Intraperitoneal glucose tolerance test

After fasting for 6 h, a 20% glucose solution was injected intraperitoneally (1 mg/g mouse weight). Blood glucose levels were measured using a glucometer (Roche Diagnostics, Meylan, France) on a drop of blood taken from the tail at times *t* = −30 min (before glucose injection), 0, 30, 60, and 90 min (after injection).

### Inflammation tests

#### Blood count (CBC)

30 µL of blood was collected from the retro-orbital sinus with a pasteur pipette previously heparinised (100 U/mL). The blood count was obtained with the Micro60S blood analyzer.

#### Flow cytometry

All operations are performed at 4 °C. The spleen and cervical lymph nodes were removed and crushed in RPMI 10% Fetal Calf Serum to obtain a cell suspension. Red blood cells were lysed in the spleen suspension using a hypertonic solution (ACK: Ammonium Chloride Potassium). The cell suspensions were centrifuged (1 500 r/min 10 min) and the cells are transferred to RPMI. To avoid non-specific labeling and to block immunoglobulin Fc receptors on leukocytes, the cells were incubated in blocking buffer (containing decomplemented rat serum, decomplemented mouse serum and anti-CD16/CD32 (BD) antibody) for 20 min, followed by 30 min with specific antibodies to anti-MHCII (V450) at 1:600 (eBioscience), anti-CD45 (BV510) at 1:100 (BD), anti-TCR (PeCy7) at 1:400 (BD), anti-CD4 (APC-Cy7) at 1:400 (eBioscience), anti-CD3 (FITC) at 1:100 (BD), and anti-CD19 (PerCpCy5. 5) at 1:200 (BD).

Cells were then fixed and permeabilised (eBioscience buffer) for 60 min at 4 °C and then labeled with anti-RORγt (APC) at 1:200 (eBisocience) and anti-Foxp3 (PE) at 1:200 (eBioscience). In parallel, isotype controls were used to identify non-specific labeling. Labeled cells were then analyzed by flow cytometry (LSR Fortessa, Becton Dickinson) and data were analyzed using FlowJo software.

To quantify cytokine production by CD4 T cells, cell suspensions were stimulated for 4 h at 37 °C with phorbol 12-myristate 13-acetate (PMA) 1/2 000 (Sigma), ionomycin 1/2 000 (Sigma) and 2.5 µg/mL Brefeldin A (Sigma). The cells were labeled with surface antibodies as described above. The cells were then fixed and permeabilised for 1 h at room temperature and finally labeled with anti-IL17 (FITC) at 1/200 (eBioscience) and anti-IL22 (PE) at 1/200 (eBioscience) for 60 min at 4 °C.

#### Real-time quantitative PCR analysis for adipose tissue

The RNA of the tissues was extracted individually using TriReagent, according to the manufacturer’s instructions. Quantification analysis of total RNA was performed by analyzing 1 µL of each sample in Nanodrop. cDNA was prepared by reverse transcription of 500 ng total RNA using a RT iScript kit (Biorad). Real-time PCR was performed with the QuantStudio™ 5 Real-Time PCR System (Thermofisher) using SYBR Green Real-Time PCR Master Mixes (Biorad) for detection, according to the manufacturer’s instructions. 36B4 was chosen as the housekeeping gene. All samples were performed in duplicate, and data were analyzed according to the 2ΔΔCT method. The identity and purity of the amplified product were assessed by melting curve analysis at the end of amplification. The expression of the genes encoding for the following proteins were analyzed : Tnfa, Serbp1, Il1b, Il10, Il6, Tgfb1. The reverse and forward primers were design by Biorad (ENTEROSYS, Labège, France).

#### DNA extraction

Adipose tissue samples were transferred on dry ice to the Vaiomer Lab in Labège, France. Genomic DNA (gDNA) was extracted using an optimized tissue-specific technique as previously described.^22^ This technique was carefully designed to maximize the recovery of bacterial DNA and to minimize any risk of contamination from the environment and the reagents. The quality and quantity of extracted gDNA were monitored by gel electrophoresis (1% w/w agarose in 0.5× TBE buffer) and NanoDrop 2000 UV spectrophotometer (ThermoFisher Scientific). All gDNA samples were stored at −20 °C until further processing.

#### Bacterial 16S rRNA sequencing

Profiling of the adipose microbiome was performed at VAIOMER (Labège, FRANCE).The V3–V4 hypervariable regions of the 16S rRNA were amplified by PCR using universal primer Vaiomer 1 F and Vaiomer 1 R. The first PCR reaction was carried out on a Veriti Thermal Cycler (Life Technologies) as follows: an initial denaturation step (94 °C for 10 min), 35 cycles of amplification (94 °C for 1 min, 68 °C for 1 min and 72 °C for 1 min) and a final elongation step at 72 °C for 10 min. Amplicons (467 bp on the Escherichia coli reference genome) were then purified using the magnetic beads CleanNGS for DNA clean-up (CleanNA). A second PCR reaction for sample multiplexing was performed using tailor-made 6-bp unique index sequences. This second PCR step was run as follows: an initial denaturation step (94 °C for 10 min), 12 cycles of amplification (94 °C for 1 min, 65 °C for 1 min, and 72 °C for 1 min) and a final elongation step at 72 °C for 10 min. Amplicons were purified as described for the first PCR round. All libraries were pooled in the same quantity to generate an equivalent number of raw reads and were sequenced on a MiSeq Illumina platform (2 × 300 bp paired-end MiSeq kit v3, Illumina).

#### Anti-*Pg* antibodies measurement

Immunoglobulin G antibodies specific to LPS of *Pg* were measured using a home-made ELISA. The wells of 96-well flat-bottom microtiter plates were coated in triplicates with LPS of *Pg*. After washing and blocking the plates, serum samples were added to individual wells, and specific mouse IgG antibodies were detected with an alkaline phosphatase-conjugated antimouse immunoglobulin. The absorbance was read at 405 nm using an ELISA plate reader. The results were expressed as an ELISA index (EI), which was the mean OD at 405 nm of a given serum sample divided by the mean OD at 405 nm of the calibrator.

### Statistical analyses

Statistical analyses were performed using GraphPad Prism V.5.00 for Windows Vista (GraphPad Software, San Diego, California, USA) by ABBIA Toulouse company France.

The results were expressed as mean values ± standard deviation (S.D). Statistical differences were measured using Two way ANOVA followed by Bonferroni post-test. A *p* value of less than 0.05 was considered significant.

For Fig. 1, we analyzed OTU counts table obtained from 94 AP patients’ granuloma microbiota; the resulting sequencing cluster’s percentages were first estimated for each patient. In the present data, we could detect the *Porphyromonas gingivalis* (*Pg*) species into one specific Porphyromonas multi-affiliation cluster, we selected thus 10 patients presenting at least 1% of the *Pg* cluster abondance from a total of 339 observed OTU clusters. The observed species counts were then extracted and ordered from the taxonomic detailed OTU profiles of the selected patients using a dedicated Python script. We classified in a next step the samples into 245 species respecting the full detected taxonomy affiliation i.e., starting from species up to the bacterial phylum. Consequently, the obtained final specie’s identifiers (noted Profile number in Fig. 1) were placed in an order related to the different level of taxonomy. The resulting species annotation reflected (or. respected) the complete bacterial taxonomic profile for which the closest species indexes would share, for example, at least the same genus type, and more separated spices indexes can share the same family to phylum type. In parallel, by considering the periapical index of apical periodontitis severity (PAI) from clinical set of data, we could split the patients into two groups: a Low PAI group with a PAI ≤ 3 noted LPAI and a high PAI group noted HPAI with PAI > 3, as respectively, the low and high severity AP patients. By applying this severity criteria to the previous selected *Pg* patients we observed a clear dominant of HPAI score into 70% of the *Pg* patients. We finally computed the median percentage of each identified species among the HPAI and LPAI groups, the two obtained median profiles were reported into Fig. 1.

To strengthen this analysis, we applied a stratified threshold-based approach to define the presence of *Porphyromonas gingivalis* (Pg) at varying abundance levels, ranging from 0.001% to 1%. This allowed us to assess the robustness of the association between Pg and lesion severity across detection sensitivities. Notably, the mean OTU detection limit across the cohort was 0.000 022 2% (±0.000 004 1%), providing sufficient resolution to support our threshold selection strategy. For example, at a 0.001% genus-level threshold, we selected 42 patients, 61% of whom had high PAI scores. At higher thresholds (0.01%, 0.1%, 1%), the proportion of high PAI patients increased progressively to 66%, 72%, and 77%, respectively, suggesting a dose–effect relationship.

In addition, we compared the association between *Pg* abundance and PAI severity across different taxonomic resolutions—genus, species, and cluster levels—each using a 1% detection threshold. This yielded 25, 23, and 10 patients respectively. These comparisons confirmed that the enrichment of *Pg* is consistently associated with more severe periapical lesions, regardless of taxonomic resolution. The proportion of patients with PAI > 3 at each level was reported to illustrate this consistency. These results are visualized in Fig. 1a–c and provide statistical support for a threshold-based microbial severity stratification model.

## Supplementary information

SuppFig1

Supp Fig 2

Supp Fig 3

Supp Fig 4

Supp Fig 5

Supplementary Figures

## Data Availability

The datasets analyzed for the present study are not publicly available due to ethical restriction, but are available from the corresponding author on reasonable request.
