# Supplementary figures and images for: Porphyromonas gingivalis-induced glucose intolerance during periapical lesions requires its LPS throught a Th17 immune response
Source: Int J Oral Sci. 2025 Nov 13;17:69. doi: 10.1038/s41368-025-00403-6 (PMC12615820; doi:10.1038/s41368-025-00403-6)

Supplementary Figure 1

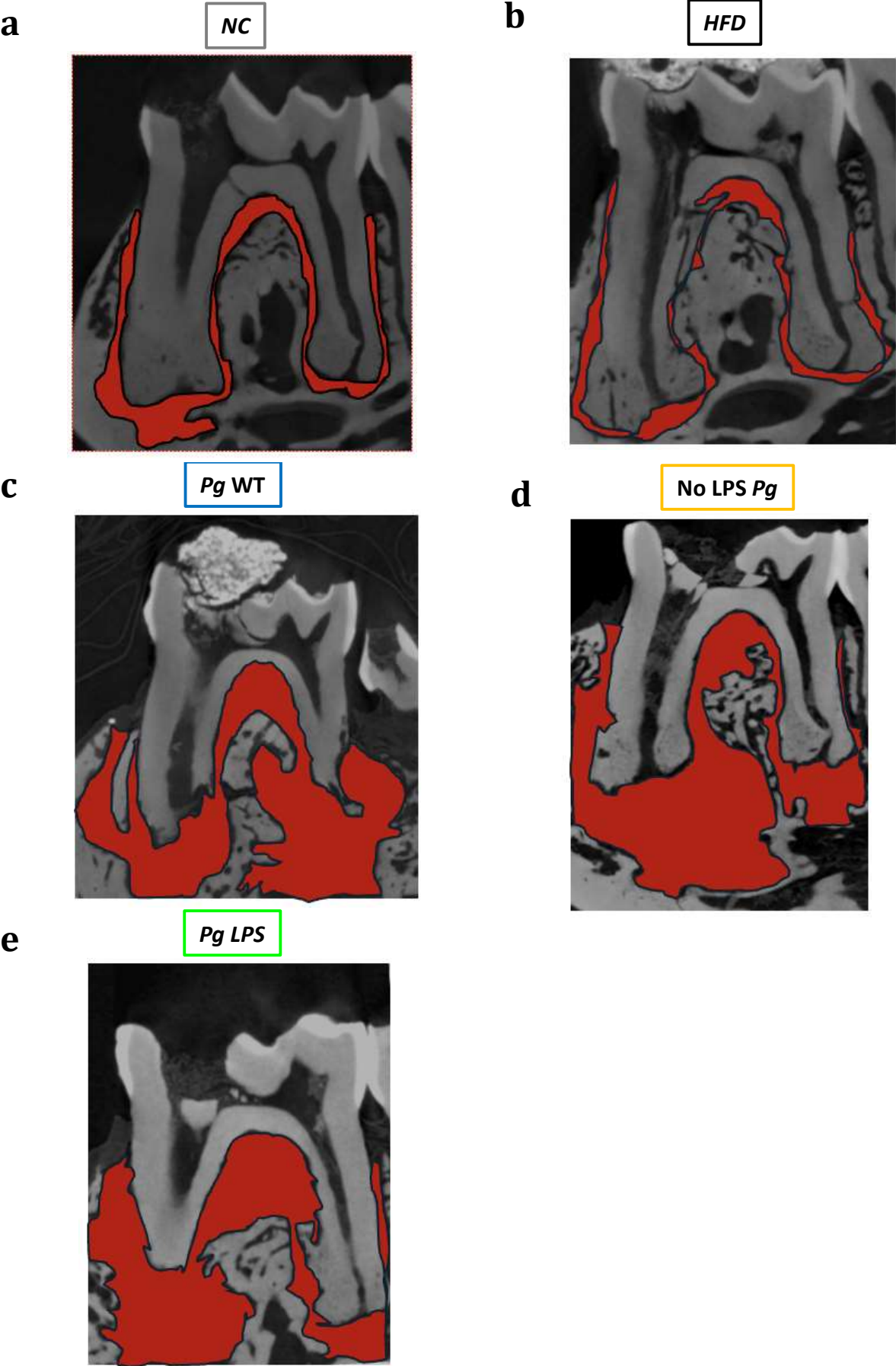

Supplement: Supplementary file 1 — SuppFig1 [file 41368_2025_403_MOESM1_ESM.pdf]

Supplementary Figure 2

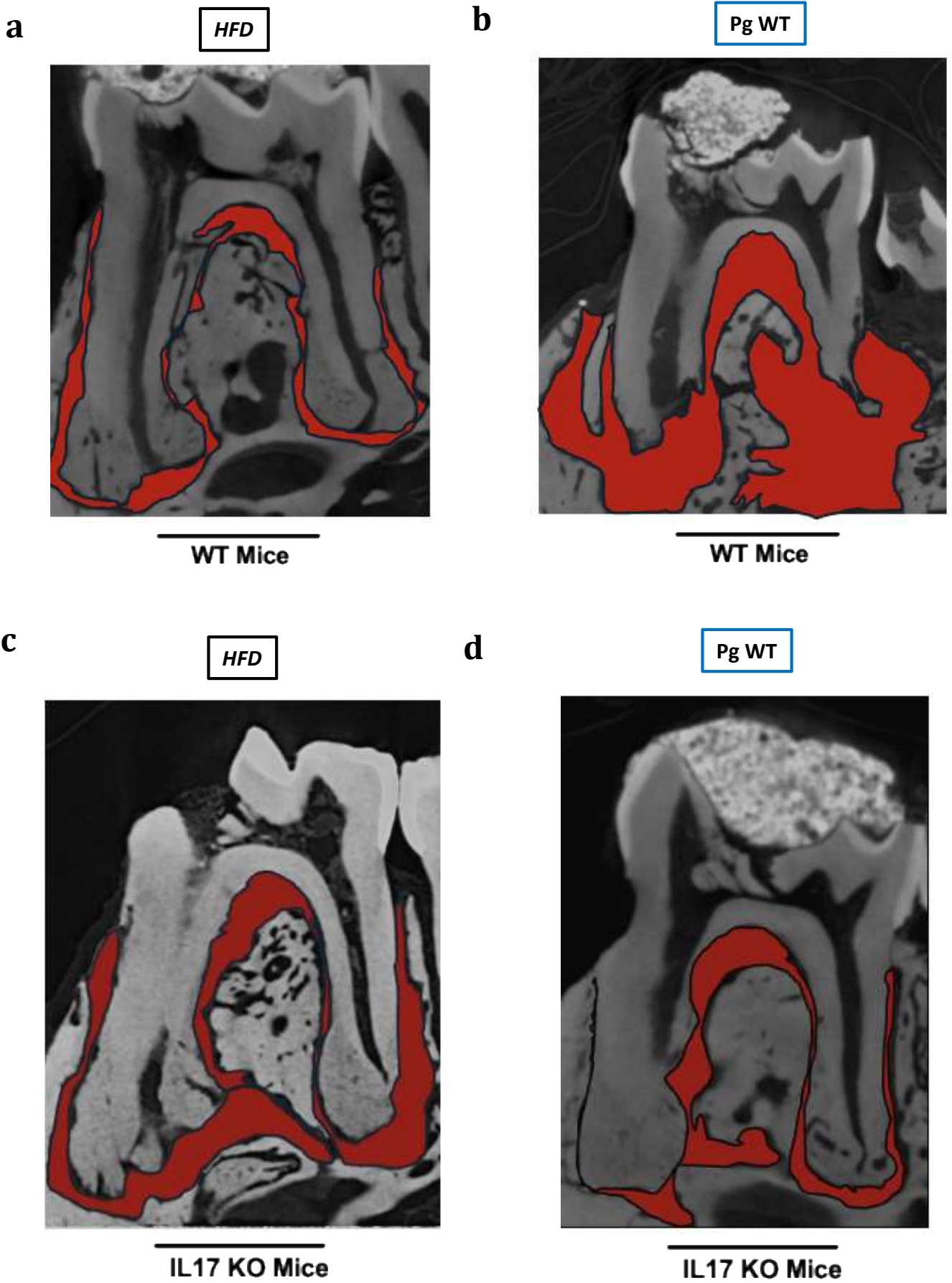

Supplement: Supplementary file 2 — Supp Fig 2 [file 41368_2025_403_MOESM2_ESM.pdf]

Supplementary Figure 5

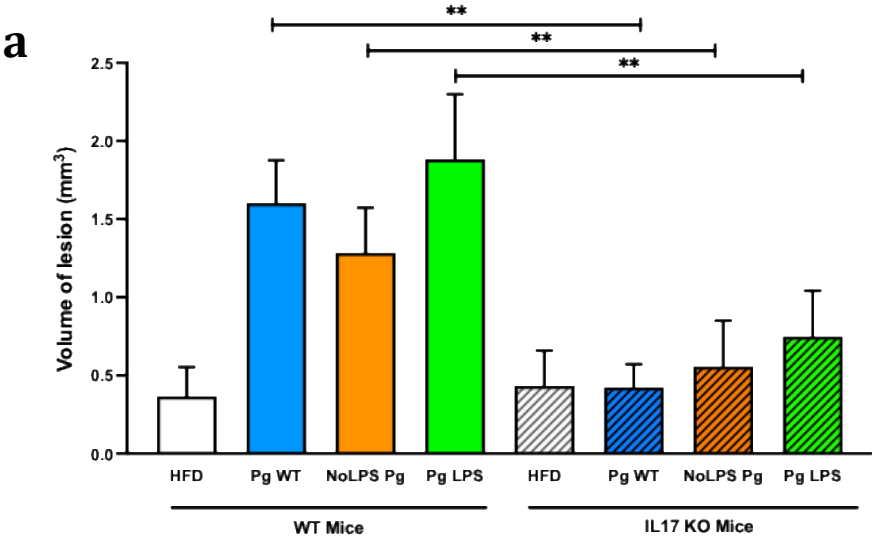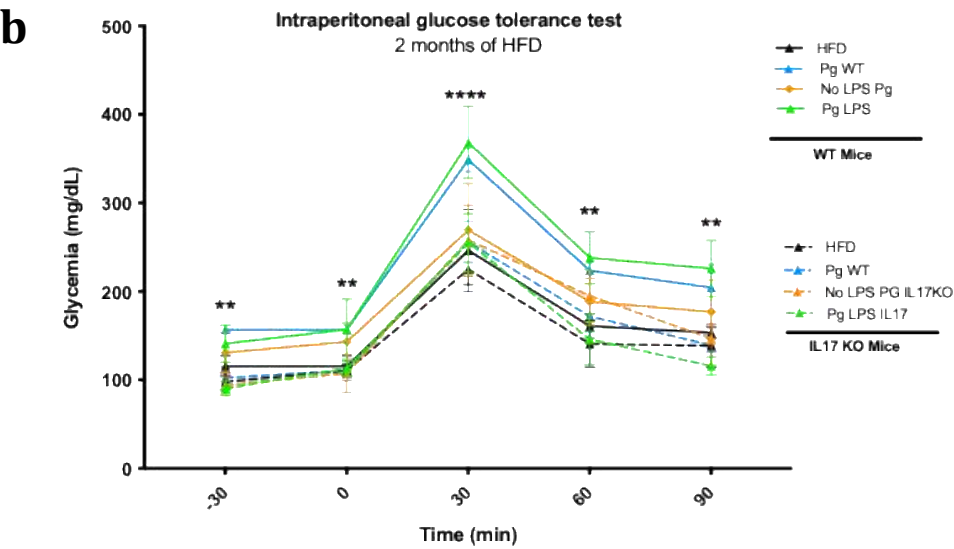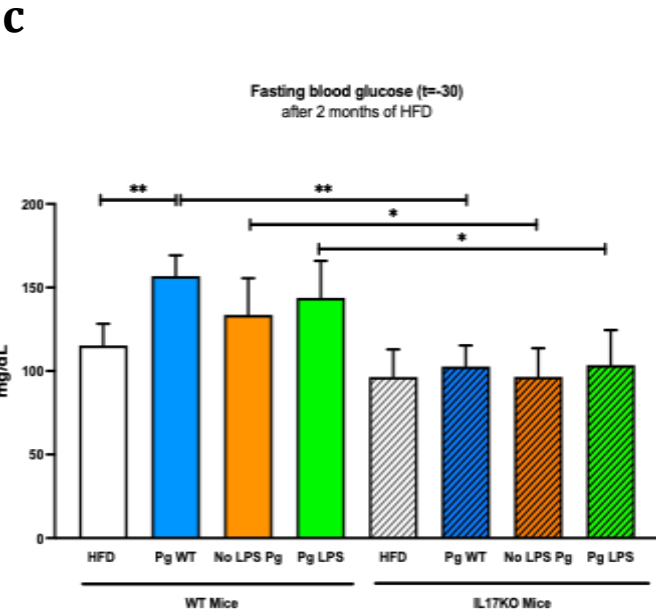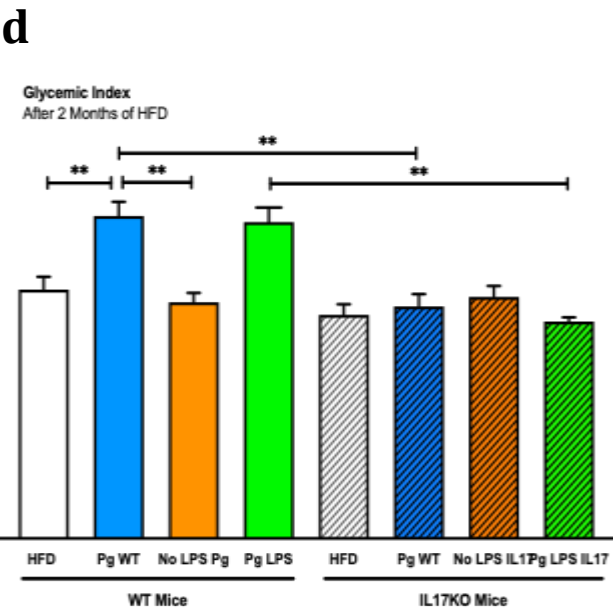

e

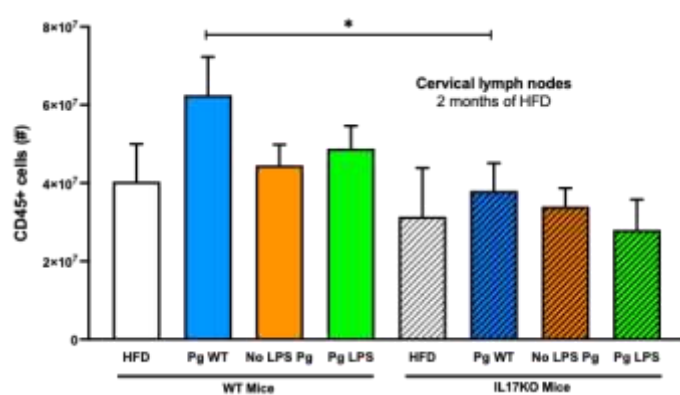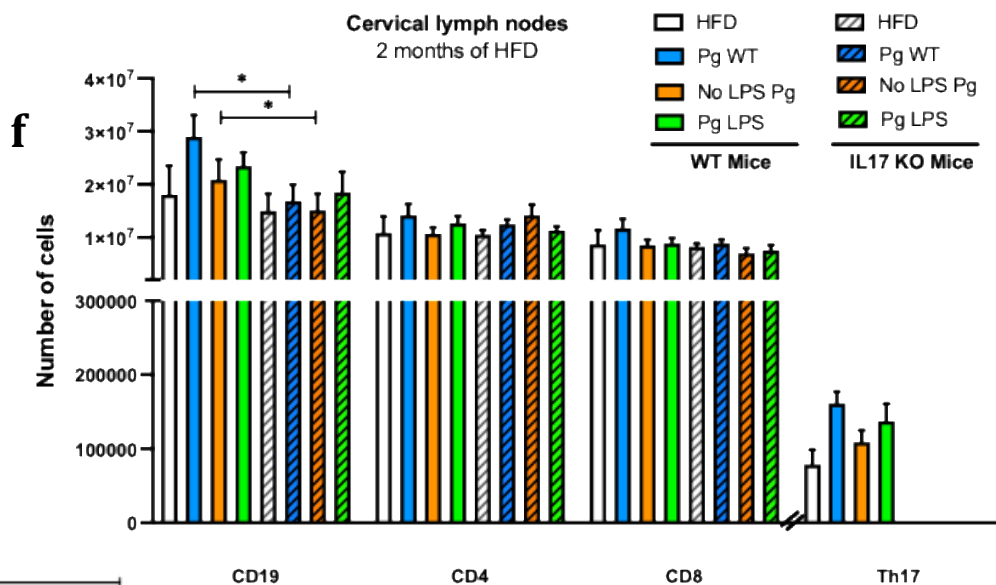

g

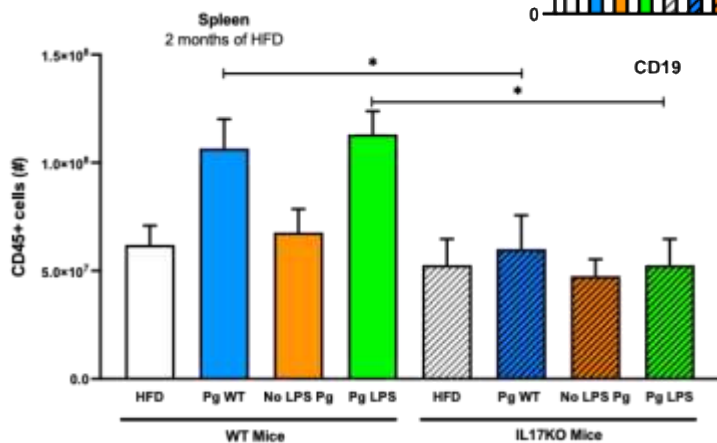

h

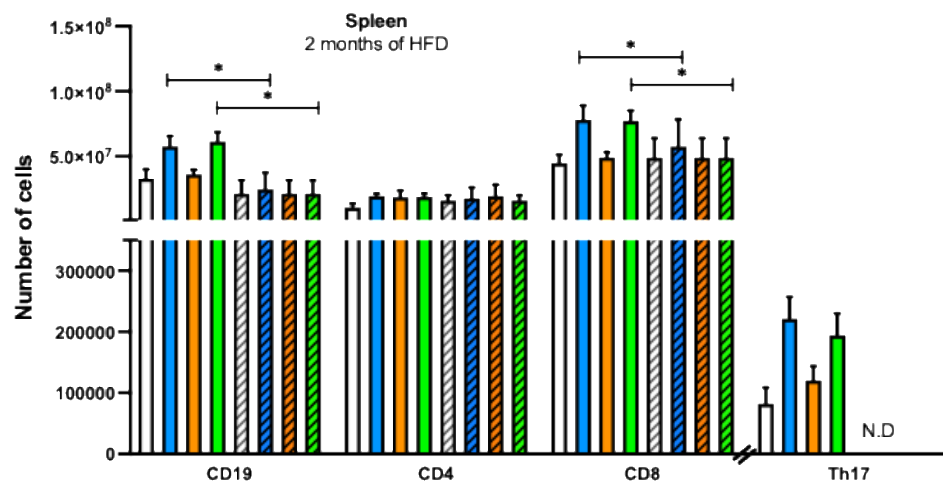

Supplement: Supplementary file 5 — Supp Fig 5 [file 41368_2025_403_MOESM5_ESM.pdf]
