# Supplementary material for: Porphyromonas gingivalis-induced glucose intolerance during periapical lesions requires its LPS throught a Th17 immune response
Source: Int J Oral Sci. 2025 Nov 13;17:69. doi: 10.1038/s41368-025-00403-6 (PMC12615820; doi:10.1038/s41368-025-00403-6)
Supplement: Supplementary file 3 — Supp Fig 3 [file 41368_2025_403_MOESM3_ESM.pdf]

Supplementary Figure 3

a

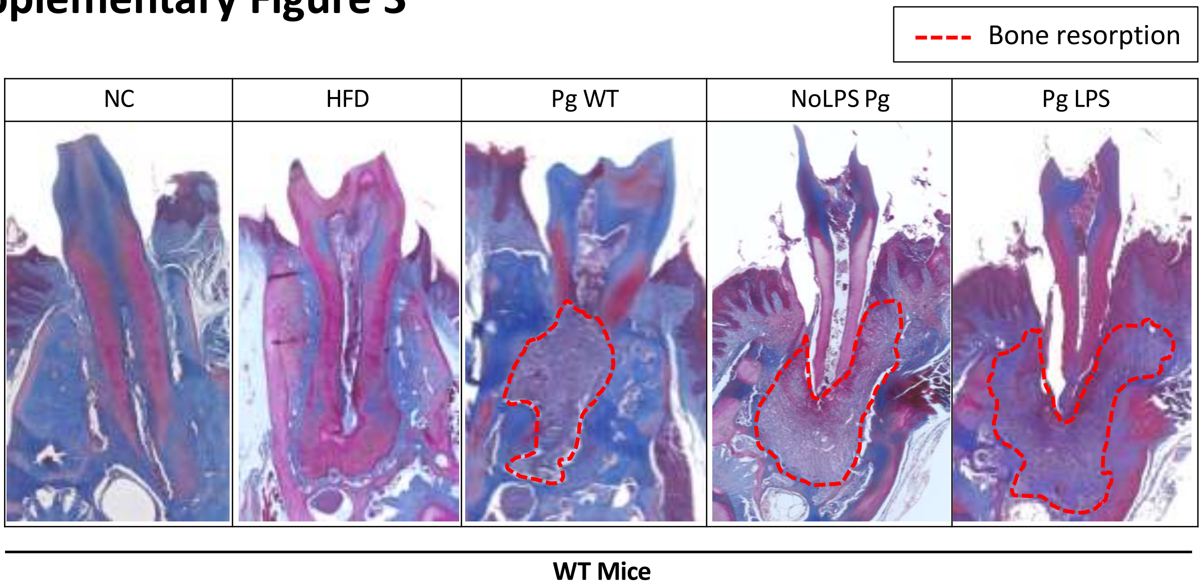

b Pg WT – WT Mice

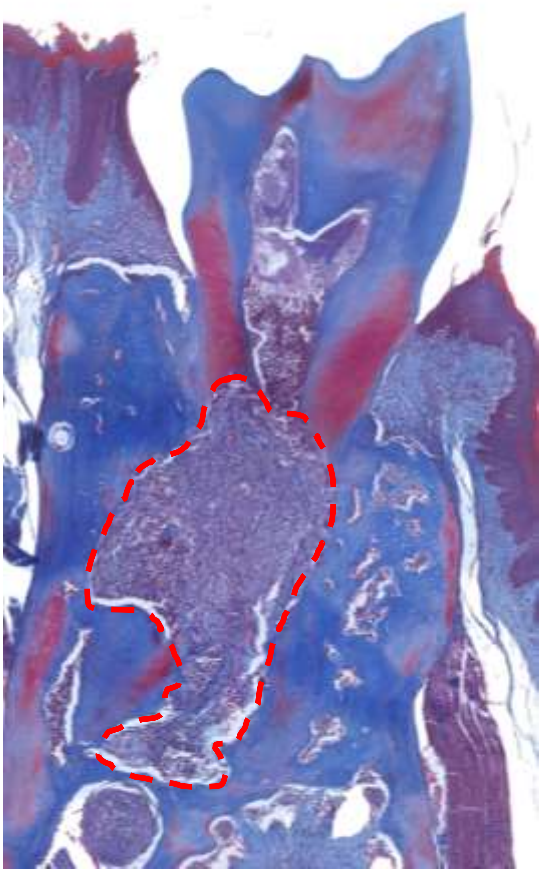

c NoLPS Pg – WT Mice

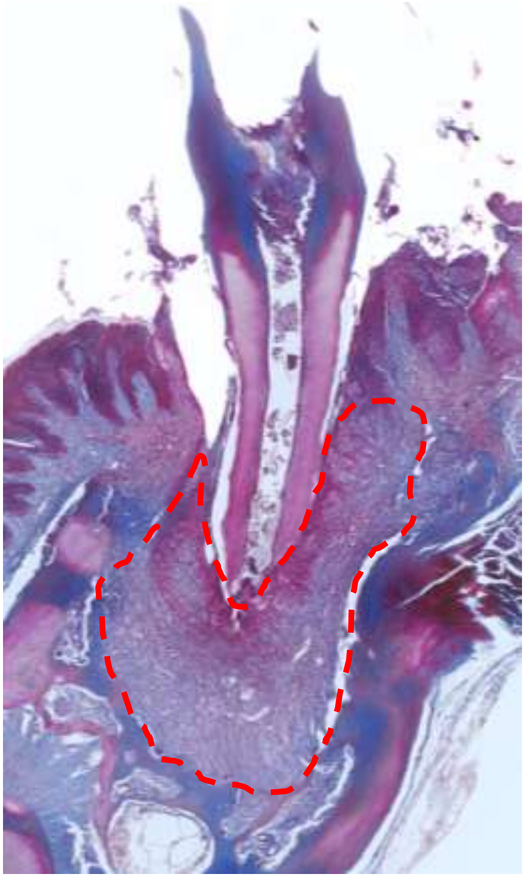

d

	Infection conditions	Inflammation	Fibrosis
WT Mice	*Pg WT*	+++	+++
	NoLPS Pg	+	+
	Pg LPS	++++	++++
