# Supplementary material for: Porphyromonas gingivalis-induced glucose intolerance during periapical lesions requires its LPS throught a Th17 immune response
Source: Int J Oral Sci. 2025 Nov 13;17:69. doi: 10.1038/s41368-025-00403-6 (PMC12615820; doi:10.1038/s41368-025-00403-6)
Supplement: Supplementary file 4 — Supp Fig 4 [file 41368_2025_403_MOESM4_ESM.pdf]

Supplementary Figure 4

---- Bone resorption

a

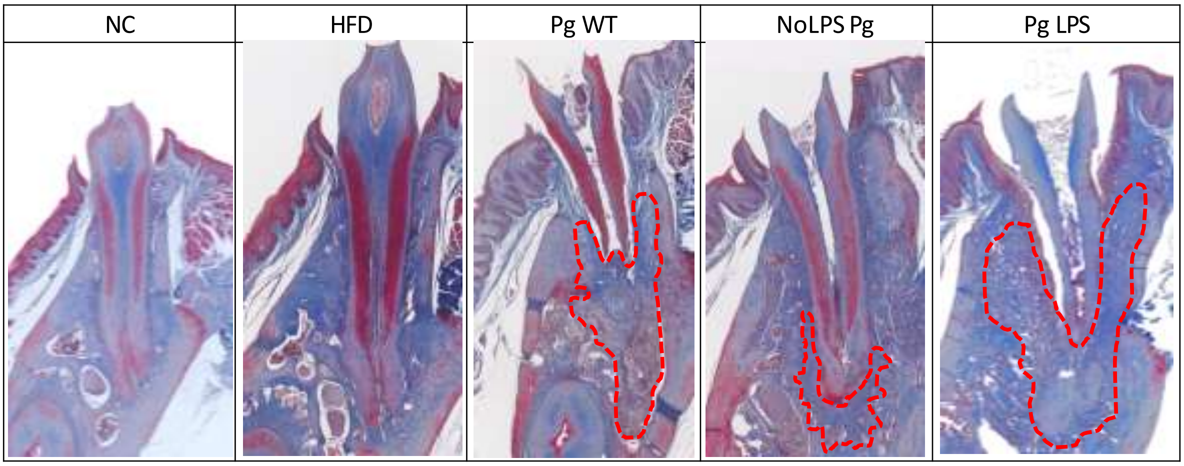

IL17 KO Mice

b Pg WT – IL17 KO Mice

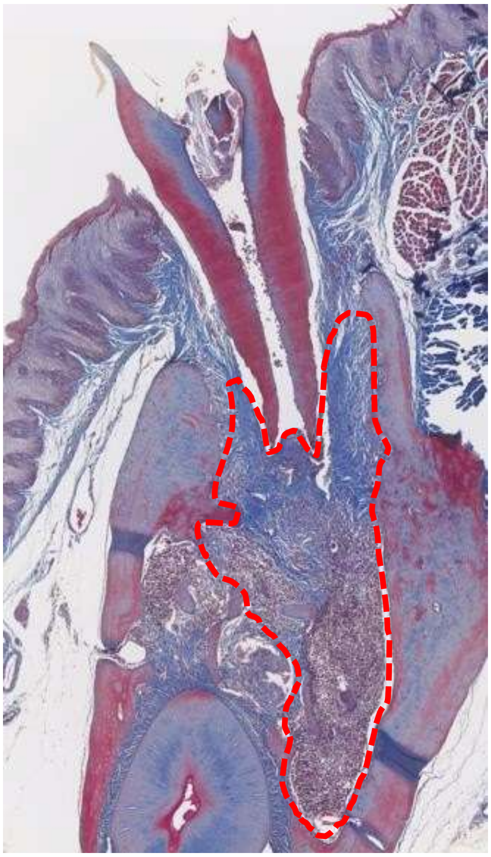

c NoLPS Pg – IL17 KO Mice

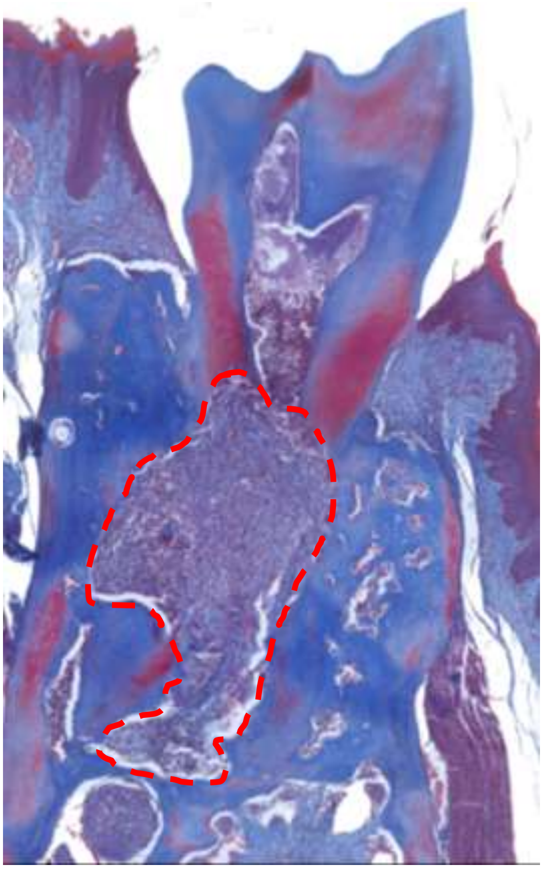

d

	Infection conditions	Inflammation	Fibrosis
IL17 KO Mice	*Pg WT*	++	++
	NoLPS Pg	-	-
	Pg LPS	++	++
