# Supplementary material for: Porphyromonas gingivalis-induced glucose intolerance during periapical lesions requires its LPS throught a Th17 immune response
Source: Int J Oral Sci. 2025 Nov 13;17:69. doi: 10.1038/s41368-025-00403-6 (PMC12615820; doi:10.1038/s41368-025-00403-6)
Supplement: Supplementary file 6 — Supplementary Figures [file 41368_2025_403_MOESM6_ESM.docx]

**Supplementary Figure 1. Representative micro-CT sagittal section of the periapical region following type of colonization.** Red areas definize the regions of interest in the apical area used to analyze the volume of periapical lesions for each conditions (Color code: a) gray = NC, b) white = HFD, c) blue = Pg WT, d) orange = No LPS Pg, e)green = Pg LPS).

**Supplementary Figure 2. Representative micro-CT sagittal section of the periapical region following type of colonization and genotype of mice.** Red areas definize the regions of interest in the apical area used to analyze the volume of periapical lesions for each conditions (Color code: a) white = HFD in WT mice, b) blue = Pg WT in WT mice, c) white = HFD in IL17 KO mice, d) blue = Pg WT in IL17 KO mice).

**Supplementary Figure 3 :Histological evaluation of periapical lesions in wild-type (WT) mice colonized with different *Porphyromonas gingivalis* (Pg) strains.** (a) Representative Masson’s Trichrome-stained sections of mandibular molars from WT mice under normal chow (NC), high-fat diet (HFD), or infected with Pg WT, LPS-deficient Pg mutant (NoLPS Pg), or LPS-expressing Pg (Pg LPS).(b–c) Magnified views showing periapical regions in Pg WT (B) and NoLPS Pg (C) conditions, highlighting inflammatory cell infiltration (red areas).(d) Semi-quantitative scoring table summarizing periapical inflammation and fibrosis across groups. Pg LPS-infected mice showed the highest degree of inflammation and fibrotic remodeling, while NoLPS Pg induced moderate changes.

**Supplementary Figure 4: Histological evaluation of periapical lesions in IL-17 knockout (IL-17 KO) mice colonized with different *Porphyromonas gingivalis* (Pg) strains.** (a) Representative Masson’s Trichrome-stained sections of mandibular molars from IL-17 KO mice under normal chow (NC), high-fat diet (HFD), or infected with Pg WT, NoLPS Pg, or Pg LPS. (b–c) Magnified views showing periapical regions in Pg WT (B) and NoLPS Pg (C) groups, showing limited inflammatory and fibrotic changes. (d) Scoring table indicating reduced periapical inflammation and fibrosis in all infected IL-17 KO mice compared to WT controls. These findings highlight the essential role of IL-17 in driving tissue pathology in response to Pg infection.

**Supplementary Figure 5. Role of *Porphyromonas gingivalis* LPS and IL-17 in periapical lesion development, glucose metabolism, and immune responses after two months of high-fat diet in WT and IL17 KO Mice.** (a) Quantification of periapical lesion volume (mm³) in WT and IL-17 KO mice. In WT mice, Pg WT and Pg LPS induced significantly larger lesion volumes compared to No LPS Pg and HFD-only groups. In IL-17 KO mice, lesion volumes remained low in all groups, including Pg WT and Pg LPS.(b) Intraperitoneal glucose tolerance test (IPGTT) curves over time following glucose injection after 2 months of HFD. WT mice colonized with Pg WT or treated with Pg LPS showed impaired glucose tolerance. IL-17 KO mice showed improved glycemic control across all conditions.(c) Fasting blood glucose levels measured at t = –30 min of the IPGTT. WT mice receiving Pg WT or Pg LPS exhibited significantly higher glycemia than control groups, while no such increase was observed in IL-17 KO mice.(d) Glycemic index calculated as the area under the curve (AUC) of the IPGTT. AUC was significantly elevated in WT mice colonized with Pg WT or treated with Pg LPS, whereas IL-17 KO mice maintained lower glycemic indices.(e) Total number of CD45⁺ immune cells in cervical lymph nodes. WT mice colonized with Pg WT or Pg LPS showed increased immune cell infiltration, while IL-17 KO mice exhibited no major variation across conditions.(f) Immune cell subset analysis in cervical lymph nodes, including CD19⁺ B cells, CD4⁺ and CD8⁺ T cells, and Th17 cells after stimulation. A significant increase in Th17 cells was detected in WT mice colonized with Pg WT or Pg LPS, and Th17 cells were absent in IL-17 KO mice.(g) Total number of CD45⁺ immune cells in the spleen. WT mice treated with Pg LPS displayed the highest immune cell counts, while responses were blunted in IL-17 KO animals.(h) Splenic immune cell subset analysis. Increased numbers of CD19⁺ and CD8⁺ cells were observed in WT mice exposed to Pg WT or Pg LPS. Th17 cells were undetectable in IL-17 KO mice, confirming the knockout model.Color code: White = HFD only (WT), Blue = Pg WT (WT), Orange = No LPS Pg (WT), Green = Pg LPS (WT),Striped white= HFD only (IL-17 KO), Striped blue = Pg WT (IL-17 KO), Striped orange = No LPS Pg (IL-17 KO), Striped green = Pg LPS (IL-17 KO).Data are presented as mean ± SD. Statistical significance: **P < 0.05, **P < 0.01, ***P < 0.001, ****P <0.000 1.* Statistical tests used: Wilcoxon–Mann–Whitney for (a), (c), (d), (e), (g), (h); two-way ANOVA with Bonferroni post-test for (b) and (f).
